# Research on the Cooperative Detection of Stochastic Resonance and Chaos for Weak SNR Signals in Measurement While Drilling

**DOI:** 10.3390/s21093011

**Published:** 2021-04-25

**Authors:** Yi Yang, Fei Li, Nan Zhang, Aiqing Huo

**Affiliations:** School of Electronic Engineering, Xi’an Shiyou University, Xi’an 710065, China; yiyang@xsyu.edu.cn (Y.Y.); 190610@xsyu.edu.cn (N.Z.); aqhuo@xsyu.edu.cn (A.H.)

**Keywords:** measurement while drilling, weak signal detection, second-order Duffing system, stochastic resonance, chaos detection

## Abstract

In the process of drilling, severe downhole vibration causes attitude measurement sensors to be erroneous; the errors will accumulate gradually during the inclination calculation. As a result, the ultimate well path could deviate away from the planned trajectory. In order to solve this problem, this paper utilized the stochastic resonance (SR) and chaos phase transition (CPT) produced by the second-order Duffing system to identify the frequency and estimate the parameters of the signal during measurement while drilling. Firstly, the idea of a variable-scale is introduced in order to reconstruct the frequency of the attitude measurement signal, and an SR frequency detection model based on a scale transformation Duffing system is established in order to meet the frequency limit condition of the SR. Then, an attitude measurement signal with a known frequency value is input into the Duffing chaos system, and the scale transformation is used again to make the frequency value meet the parameter requirement of chaos detection. Finally, two Duffing oscillators with different initial phases of their driving signal are combined in order to estimate the amplitude and phase parameters of the measurement signal by using their CPT characteristics. The results of the laboratory test and the field-drilling data demonstrated that the proposed algorithm has good immunity to the interference noise in the attitude measurement sensor, improving the solution accuracy of the inclination in a severe noise environment and thus ensuring the dynamic stability of the well trajectory.

## 1. Introduction

In the process of dynamic measurement while drilling (MWD), there are complex noises in the attitude measurement signal due to the strong vibration of bottom-hole drilling assemblies, which are produced by the bit–rock interaction and the collision between the drill string and the borehole wall. On the one hand, the frequency range of the noise presents the characteristics of wideness and randomness, which are shown in Table 1; on the other hand, the lateral vibration acceleration of the near-bit during drilling is generally about 10 g or up to 30 g [1], while the amplitude of the useful signal is generally less than 1 g. Therefore, the signal-to-noise ratio (SNR) of the attitude measurement signal is usually as low as −20 dB, or even lower. Thus, the useful signal is annihilated in the strong vibration background noise, which negatively interferes with the tool attitude measurement and makes the MWD invalid [2,3]. Multi-frequency, high amplitude noise interference and extremely low SNR are always the technical difficulties in the field of the dynamic measurement of steerable drilling tools.

At present, the commonly used detection method for underground weak SNR signals is a digital filter based on time-frequency analysis, the premise of which is that the spectrum of useful signal and noise does not overlap, the useful signal can be retained, and the irrelevant noise components can be filtered in the filtering process [4,5]. However, in the collected attitude measurement signals, due to the variety of interference sources, the frequency distribution of the noise signal is very complex, as shown in Table 1 [6], which is bound to be close to the frequency of a useful signal. Therefore, while suppressing the noise, the useful signal will inevitably be suppressed or damaged [7]. Even because of the complex and changeable strong noise interference, it is difficult to detect the weak SNR signal which overlaps with the noise spectrum, which leads to the invalidity of MWD.

Because of the limitations of the traditional detection method of weak signals, it is necessary to explore a new detection method for weak SNR signals in the pit. In recent years, the intensive study of nonlinear science has provided a new idea to understand, analyze and solve the problem of weak signal detection. Chaos is a special form of nonlinear dynamic system. Since the 1990s, many scholars have applied chaotic oscillators to weak signal detection, and have achieved remarkable results [8,9,10,11]. Moreover, the emergence of memristor has made weak signal detection based on chaotic oscillator enter a new stage of research [12]. As a kind of nanodevice with memory and nonlinear characteristics, memristor is conducive to the generation of a high-frequency chaotic oscillation signal with complex dynamic characteristics [13]; as such, it has wider application prospects in image encryption [14], secure communication [15] and other fields. In addition, the nonlinear system will produce a stochastic resonance (SR) phenomenon under the appropriate parameter conditions [16]. Scholars have deeply studied this phenomenon, and have applied it to underwater acoustic communication [17], fault diagnosis [18,19] and Internet of Things communication [20]. The experimental results show that this method can successfully detect the weak characteristic signal in strong noise or a complex noise background.

Through the above research, the SNR of the signal which was detected by chaos and SR methods is much lower than that of the traditional detection methods. As such, it has wider application prospects. In many nonlinear systems, the second-order Duffing system, which has both SR effects and chaotic phase transition (CPT) characteristics, has become a research hot spot in the field of weak signal detection.

SR refers to the phenomenon by which, under the synergistic effect of input signal and noise, the SNR of the output signal increases first and then decreases with the increase of noise by using a nonlinear system, and the peak value appears at a certain noise intensity, resulting in resonance output [21,22]. At present, there are few reports about the application of SR in weak periodic signal detection while drilling. The related research includes Zhang et al. [23], who proposed an adaptive optimization method of SR parameters based on a genetic simulated annealing algorithm for mud pulse signal detection under the complex background of MWD, and realized signal detection under low SNR. Based on the noise energy collection mechanism of SR, Park et al. [24] transformed the high-intensity and low-speed vibration noise generated by drilling tools in the process of drilling. The results show that this method can effectively identify the frequency of the signal to be measured. According to the Langevin equation, Chen [25] established the SR detection model of a down-hole acoustic communication signal, selected appropriate parameters, and finally obtained the frequency value of the useful signal.

Through the above research, it is not difficult to see that using the SR effect produced by the nonlinear system under the condition of appropriate parameters can effectively improve the SNR of the system output signal, identify the frequency of the down-hole useful signal, to realize the detection of a weak signal. However, SR cannot measure the ‘size’ of the measured signal, that is, the amplitude and phase parameters of the signal, so the complete information of the down-hole attitude measurement signal cannot be obtained.

Using the sensitivity of the CPT to system parameter perturbation and its noise immunity, it can achieve extremely low SNR detection in the case of fewer measurement data points and arbitrary colored noise background [26,27]. Therefore, compared with the traditional detection method, this method can detect the signal with lower SNR, and is not limited by the statistical characteristics of the noise [28,29].

Wu et al. [30] designed an improved Duffing chaotic system and applied it to the resistivity detection of a near-bit electromagnetic logging signal. The test results showed that the smaller the amplitude of the tested signal, the more sensitive and noise resistant the system is to the signal, and the lower the SNR of the detectable signal. At present, the research on the application of a second-order Duffing system to the weak signal detection of dynamic MWD is under-developed, and the current research results have certain limitations in their application: they are affected by the strong vibration near the bit, and the frequency of the downhole attitude measurement signal constantly fluctuates based on the rotation speed of the drilling tool, rather than being fixed. Therefore, it cannot be directly used as an input signal and linear superposition with the system driving term. Although the scale transformation method is used to solve this problem in [31], the method requires the high accuracy of frequency matching, so it will reduce the accuracy of the frequency detection in a field-drilling application, and it is not easy to meet the real-time requirements of MWD.

In order to overcome the shortcomings and limitations of SR and chaos detection, a new detection scheme for weak SNR signals in dynamic MWD is investigated in this paper. The scheme takes the second-order Duffing system as the theoretical framework, and uses the synergy of SR and CPT to obtain the complete parameter information of the useful signal. It not only avoids the matching requirement of the frequency of the measured signal in chaos detection but also makes up for the deficiency that the signal “size” cannot be measured by SR. The simulation test was carried out on the vibration platform system and the field-drilling data of a well in northern Shaanxi, which verified the feasibility and efficiency of the proposed algorithm being applied to down-hole weak SNR signal detection.

## 2. Frequency Detection of a Weak Signal Based on the SR of a Variable-Scale Duffing System

### 2.1. Basic Principle of Frequency Detection Based on the SR of a Duffing System

Consider a second-order Duffing system driven by useful signal *s*(*t*) and noise signal *n*(*t*), as follows:

(1) $$x^{\prime \prime }+kx^{\prime }-ax+bx^{3}=s(t)+n(t)$$

where *k* is the damping ratio, *a* and *b* are the system parameters, *x* is the system output of the Duffing oscillator, and *f*(*x*) = −*ax* + *bx*^3^ is the nonlinear restoring force.

When *s*(*t*) = 0 and *n*(*t*) = 0, the system potential function of the second-order Duffing system is as follows:

(2) $$U(x)=-\frac{1}{2}ax^{2}+\frac{1}{4}bx^{4}$$

The relationship between the potential function of the system and the nonlinear restoring force is as follows:

*U*′(*x*) = *f*(*x*) (3)

**Figure 1:**
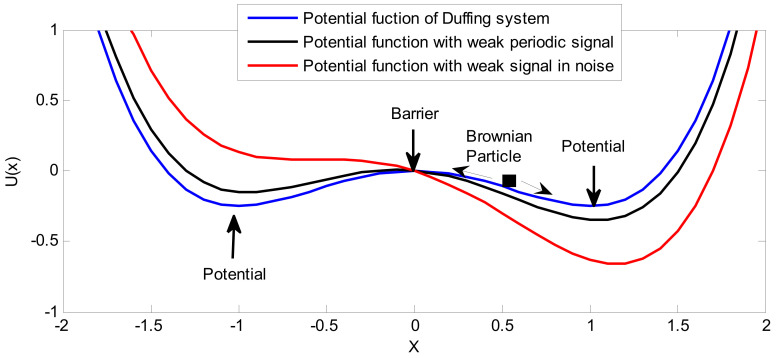
SR mechanism of the second-order Duffing System.

The system potential function is shown as the blue curve in Figure 1.

It can be seen that Equation (2) is a typical bistable potential field structure. Let *du*(*x*)/dx = 0; the equilibrium point of the system can be found as follows:

(4) $$\left\{\begin{array}{l} x_{m1,m2}=\pm \sqrt{a/b}\\ x_{n}=0 \end{array}\right.$$

where *x_m_*_1_ and *x_m_*_2_ are the stable equilibrium points, and *x_n_* is the unstable equilibrium point. Thus, two potential wells separated by the middle barrier are formed. The height difference between the barrier and the well is easy to find, as follows:

(5) $$∆U=U(x_{n})-U(x_{m})=0-\left(-\frac{a^{2}}{4b}\right)=\frac{a^{2}}{4b}$$

Under the above conditions, Brownian particles will eventually fall into one of the potential wells after a transient process. The specific potential well is determined by the initial conditions of the system. Therefore, the second-order Duffing system is also called a bistable system.

Let the useful signal be *s*(*t*) = *A*cos(2π*f*_0_*t* + *φ*) and the noise signal be *n*(*t*) = 0; then, the system potential function is periodically modulated by the useful signal, and changes from *U*(*x*) to *V*(*x*), as follows:

(6) $$V(x)=U(x)-xA\cos(2\pi f_{0}t+\varphi )=-\frac{ax^{2}}{2}+\frac{bx^{4}}{4}-xA\cos(2\pi f_{0}t+\varphi )$$

In this way, the two potential wells of the potential function are periodically raised or deepened, as shown in the black curve in Figure 1. At this time, there is a critical amplitude *A*_c_. When *A* < *A_c_*, Brownian particles will cause local periodic motion near a potential well; when *A* > *A_c_*, Brownian particles will cause large-scale periodic transition motion between two potential wells. The critical amplitude *A_c_* means that *V*(*x*) changes from a bistable structure to a monostable structure when *s*(*t*) is at its maximum or minimum, and its theoretical value is as follows:

(7) $$A_{c}=\sqrt{\frac{4a^{3}}{27b}}$$

When the useful signal *s*(*t*) and noise *n*(*t*) exist at the same time and the system parameters are appropriate, Brownian particles will cause a large-scale transition between the two potential wells with the help of noise, even if *A* < *A_c_*. At this time, the signal, noise, and system achieve synergy, and noise will have a positive effect, such that part of the noise energy is transferred to the useful signal. The signal energy is enhanced, the output SNR of the system reaches its maximum, and the SR is realized, as shown in the red curve in Figure 1.

When the second-order Duffing system produces the SR effect, there will be an obvious peak at the frequency of the signal *s*(*t*), which can be used to detect the frequency value of the weak SNR signal in the strong noise background. This is the basic principle of using the SR characteristics of the Duffing system to detect the frequency value of a weak signal.

Although the SR characteristics of the Duffing system can effectively identify the frequency value of a weak signal with noise interference, there are still limitations when it is applied to the detection of a down-hole attitude measurement signal: the SR of a Duffing system based on adiabatic approximation theory is only applicable to the condition of a small frequency parameter (near 0.01 Hz). However, in the process of MWD, the frequency of the useful signal is mainly determined by the rotation speed of the drill string, which varies from 1 to 3 Hz during normal drilling, which is beyond the limit of Duffing system SR characteristics on the frequency parameters. In this case, the SR phenomenon cannot be obtained by direct numerical calculation, and the useful signal in the noise cannot be identified.

### 2.2. Frequency Detection Based on the SR of a Variable-Scale Duffing System

In order to obtain the SR phenomenon through the Duffing system under the condition of large frequency parameters, this section uses the step transformation method to improve the standard Duffing model, which was shown in Equation (1). Furthermore, let the measured signal *s*(*t*) be as follows:

*s*(*t*) *= λ*cos(2*πf*_0_*t + φ*) (8)

where *f*_0_ is the actual frequency value of the measured signal.

Keeping the other parameters of the Duffing system unchanged, a variable step-size coefficient *R* is introduced in order to enlarge *s*(*t*) by *R* times on the time axis, namely *t*′ = *Rt*. In this case, if the variable step coefficient *R* = 100*f*, the measured signal can be expressed as

(9) $$s(t^{\prime })=\lambda \cos(2\pi ft+\varphi )=\lambda \cos(2\pi \cdot 0.01R\cdot t^{\prime }/R+\varphi )=\lambda \cos(2\pi \cdot 0.01t^{\prime }+\varphi )$$

It can be seen from Equation (9) that the frequency of the measured signal is compressed from the actual value to 0.01 Hz on the frequency axis. Under the action of the Duffing system, the existence of signal *s*(*t*′) can be detected, and then the frequency value of the actual measured signal can be determined according to the relationship of *R* = 100*f*. However, when processing the actual measured signal, it is impossible to directly transform the collected sensor signal through a linear transformation, such as Equation (9). Therefore, the step size of the numerical calculation is transformed in order to realize frequency parameter reconstruction. The specific steps are as follows.

Step 1: Assuming that the sampling frequency is *f_s_*, the step size of the numerical calculation should be as follows:

*dt* = 1/*f_s_* (10)

Step 2: By introducing the variable step-size coefficient *R*, the numerical calculation step size becomes

(11) $$dt^{\prime }=Rdt=\frac{R}{f_{s}}$$

At this time, the signal time interval is increased by *R* times, and the corresponding signal frequency is compressed by *R* times. In other words, a down-hole attitude measurement signal with a sampling frequency of *f*_S_ and an actual frequency of *f* is transformed into a signal with the sampling frequency of *f*_S_/*R* and a characteristic frequency of *f*/*R* through step transformation.

Step 3: Input the signal after the step transformation into the Duffing system, and make the calculation step meet Equation (11); complete the parameter reconstruction of the MWD signal, and then identify the frequency value of the weak SNR signal through SR, which was produced by the Duffing detective model in Equation (1).

It can be seen from the above analysis that the step size transformation does not change the value of the measured signal, but only changes the frequency or time scale of the signal, compresses or amplifies it on the time axis, and reorders the values, which will not affect the final calculation results.

**Figure 2:**
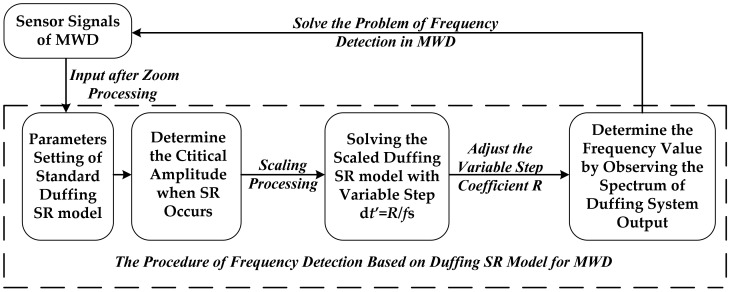
The procedure of frequency detection for MWD based on the SR of a variable-scale Duffing system.

It can be seen from the above variable-scale Duffing system detection principle that no matter what the frequency parameter of the measured signal is, it is reduced to 0.01 Hz. The frequency of the attitude measurement signal is compressed by step transform coefficient *R* to meet the parameter condition of SR frequency detection. The process of frequency detection based on the SR phenomenon of the variable-scale Duffing system for the attitude signal in MWD is shown in Figure 2.

## 3. Parameter Estimation of a Weak Signal Based on the CPT of a Duffing System

In order to calculate the real-time attitude information of drilling tools, it is necessary to restore the complete signal waveform. Therefore, in addition to the frequency information extracted in the previous section, it is also necessary to obtain the amplitude and phase parameters of the useful signal. Another nonlinear property of the Duffing system, namely CPT, is used. However, the traditional chaos detection method based on the Duffing system cannot estimate multiple parameters of the signal at the same time, and the existing phase estimation method has low computational efficiency, which makes it difficult to meet the requirements of accuracy and real-time MWD. For this reason, this section proposes a method of amplitude and phase synchronization estimation that is suitable for down-hole attitude measurement signals.

For the following Holmes-type Duffing chaotic system:

(12) $$x^{\prime \prime }+0.5x^{\prime }-ax+bx^{3}=A\cos(\omega t+\alpha )+\gamma \cos(\omega t+\varphi )+n(t)$$

where *A*cos(*ωt* + *α*) is driving term; *γ*cos(*ωt* + *φ*) is input, that is, the measured signal *s*(*t*) in Equation (1); and *γ* and *φ* are the amplitude and phase of the measured signal, that is, the quantity to be calculated.

When the total amplitude of the signal at the right end of the equation changes, the state of the system goes through homoclinic orbit, chaos, and a large-scale periodic state, namely phase transition. Chaos detection obtains the amplitude and phase parameters of the measured signal through this phase transition.

In order to make the Duffing system produce the phase transition effect smoothly, the angular frequency of the driving term and input term should be set at 1 rad/s at the same time; otherwise, the dynamic characteristics of the system will deteriorate, which will bring great difficulties and errors to the detection. Therefore, based on the scale transformation theory mentioned in the previous section, the step size of the numerical calculation is transformed to reconstruct the angular frequency. Therefore, the Duffing chaotic system after the frequency transformation is obtained as follows:

(13) $$x^{\prime \prime }+0.5x^{\prime }-ax+bx^{3}=A\cos(t+\alpha )+\gamma \cos(t+\varphi )+n(t)$$

By comparing Formulae (12) and (13), it can be seen that the angular frequency of the driving term and the input term simultaneously change from *ω* to 1, where the value of *ω* is provided by the SR frequency detection in the previous section.

By simplifying the first two terms at the right end of the equal sign in Equation (13), we can obtain

(14) $$\begin{array}{ll} M(t)\; & =A\cos(t+\alpha )+\gamma \cos(t+\varphi )\\ & =\sqrt{A^{2}+2A\gamma \cos(\alpha -\varphi )+\gamma^{2}}\cdot \cos(t+\theta ) \end{array}$$

(15) $$\theta =\arctan\frac{\left(A\sin\alpha +\gamma \sin\varphi \right)}{\left(A\cos\alpha +\gamma \cos\varphi \right)}$$

It can be seen that the input term and the driver term are combined into a trigonometric function term. By observing the position of *θ* in Formula (14), we can see that the value of *θ* hardly affects the amplitude parameters of the Duffing chaotic system, but only changes the initial position of the trajectory solution. It is the amplitude term of the trigonometric function that affects the detection result. Therefore, in order to turn the Duffing system from a chaotic state to a large-scale periodic state, the equation of the *M*(*t*) amplitude term is established as follows:

(16) $$\sqrt{A^{2}+2A\gamma \cos(\alpha -\varphi )+\gamma^{2}}\geq 0.827$$

where 0.827 is the critical amplitude that can turn the system output from a chaotic state to a large-scale periodic state.

Let *A_a_* denote the amplitude of the driving term when the Duffing system changes from a chaotic state to a large-scale periodic state. As such, there will be

(17) $$\sqrt{A_{a}^{2}+2A_{a}\gamma \cos(\alpha -\varphi )+\gamma^{2}}=0.827$$

where *A_a_* can be obtained by observing the phase state of the Duffing system.

It can be seen from the equation above that when *A_a_* is determined, (16) is a binary equation with the amplitude *γ* and phase *φ* of the measured signal as variables. Therefore, we consider the use of a similar method to establish a binary equation regarding *γ* and *φ,* and then estimate the amplitude and phase of the measured signal synchronously by solving the binary function equations.

For the parameter estimation problem to be solved in this section, first let the initial phase α of the driving signal take 0 and π respectively, and input the measured signal into two Duffing chaotic systems with different α; then, adjust the amplitude of the driving signal of the two Duffing equations step by step, observe the change of the phase diagram of the system, and record the amplitude when the phase transition occurs. If *A_a_*_1_ and *A_a_*_2_ are used to represent the amplitude of the driving signal of which the output phase state changes when *α* is 0 and π respectively, then the binary quadratic equations regarding the amplitude *γ* and phase *φ* of the measured signal can be expressed as

(18) $$\left\{\begin{array}{c} \sqrt{A_{a1}^{2}+2A_{a1}\gamma \cos\varphi +\gamma^{2}}=0.827\\ \sqrt{A_{a2}^{2}-2A_{a2}\gamma \cos\varphi +\gamma^{2}}=0.827 \end{array}\right.$$

(19) $$\left\{\begin{array}{c} \gamma =\sqrt{0.827^{2}-A_{a1}A_{a2}}\\ \varphi =\arccos\left(\frac{A_{a2}-A_{a1}}{2\sqrt{0.827^{2}-A_{a1}A_{a2}}}\right) \end{array}\right.$$

**Figure 3:**
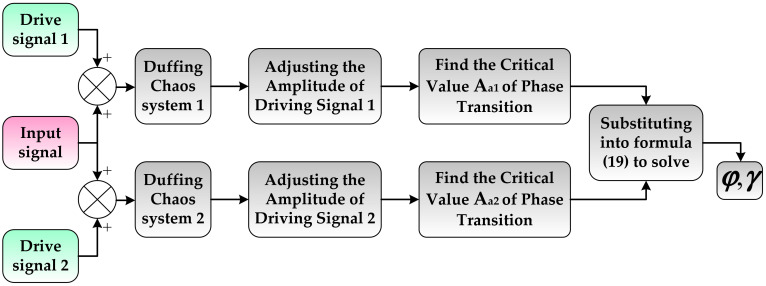
The synchronization estimation model of the amplitude and phase parameters for MWD.

Formula (19) is the estimation formula of the amplitude *γ* and phase *φ* of the measured signal. The principle of parameter synchronization estimation based on a double Duffing system is shown in Figure 3.

Based on the above analysis, the steps of the frequency detection and parameter estimation can be summarized as follows:

1. Determine the parameters of the SR model of the Duffing system. For example, in Equation (1), *k* = 0.5, *a* = *b* = 1, and the initial value of state (x(0), x′(0)) = (0, 0), and the critical amplitude *λ*_C_ = (4*a*^3^/27*b*) ≈ 0.148 are usually taken.
2. Estimate the strength of the measured sensor signal, and normalize the collected signal; that is, the amplitude of the signal is linearly compressed or expanded so that the signal strength is within the appropriate processing range, i.e., less than the critical amplitude *λ*_C_.
3. Introduce the variable-scale coefficient *R* to change the signal with the sampling frequency of *f*_s_ and signal frequency of *f*_0_ into a signal with a sampling frequency of *f*_s_/*R* and an actual frequency of *f*_0_/*R*. Take the calculation step *dt*′ = *R*/*f*_s_ to solve the Duffing SR model and obtain the system waveform and signal spectrum.
4. Adjust the variable-scale coefficient *R* and observe the spectrum of the output signal. If the signal has an obvious peak at frequency 0.01, it will produce SR effect, and the value of *R* is the frequency value of the measured signal.
5. Set the relevant parameters of the Duffing chaotic system, such as the amplitude of driving term *A* = 0.825 in Equation (12).
6. Set the initial phase of the driving term to 0 and π, and record it as detection systems 1 and 2. The measured signal is input into two detection systems respectively, and the change of the output phase diagram of the system is observed. If the output of detection system 1 is chaotic after adding the measurement signal, the amplitude of the driving signal will be increased gradually with a certain amplitude until the system output jumps to the periodic state; if the system output is periodic, the amplitude of the driving signal will be decreased gradually until the system jumps from the periodic state to the chaotic state. The amplitude of the system jumping into a large-scale periodic state is denoted as *A_a_*_1_.
7. Use the same method to obtain the amplitude of detection system 2 when it jumps to a large-scale periodic state, which is recorded as *A_a_*_2_. Finally, *A_a_*_1_ and *A_a_*_2_ are substituted into Equation (14) in order to obtain the estimated values of the amplitude and phase parameters of the MWD signal.

## 4. Performance Evaluation and Discussion

The cooperative detection method based on the SR and CPT of the Duffing system developed in this paper was verified by laboratory bench and field measurement data. A comparative analysis with the original measurement signal was conducted in order to comprehensively evaluate the performance of the proposed algorithm.

### 4.1. Laboratory Testing

In the laboratory, a strong vibration environment is simulated by using a six-degree space vibration test-bed, and a vibration interference signal with Gaussian white noise characteristics is generated. In this testing, the performance of the frequency detection and parameter recovery of the proposed method for extremely low SNR signals will be verified; as such, we do not consider other noise types. The detection effect under the colored noise background will be tested and analyzed in Section 4.2.

The rotation angular velocity of the inclinometer calibration device was set at 2π~6π rad/s (the frequency of the measured signal is between 1~3 Hz), which is consistent with the rotation state of the actual drilling tool in the process of drilling; the two parts of the signal are superimposed through the data acquisition system, and the comprehensive performance of the proposed method is verified as the test sample.

**Figure 4:**
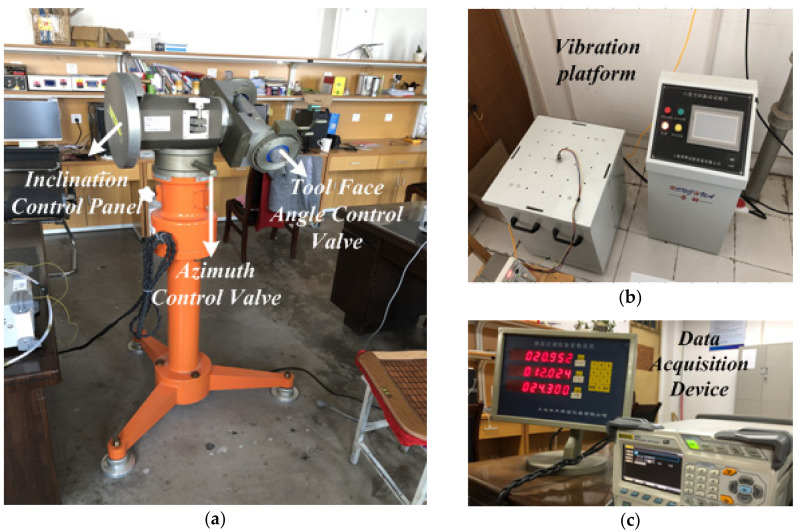
The main instruments and equipment in the laboratory: (**a**) an inclinometer adjusting device; (**b**) a six-degree space vibration experiment platform; (**c**) a data acquisition device.

The main instruments and equipment for laboratory testing included a set of TX-3S inclinometer calibration devices, the six-degree space vibration experimental platform, and the data acquisition device, as shown in Figure 4 [2]. The other selected experimental equipment included a RIGOL DS1204B oscilloscope and a DC power supply.

#### 4.1.1. Simulation Experiment of the Frequency Detection Based on SR

In order to verify the detection performance of the variable-scale Duffing SR system on weak SNR signal while drilling, the output signal of a three-axis accelerometer is obtained by using inclinometer calibration device, as shown in Figure 4a. In this section, the x-axis accelerometer signal is taken as an example for the simulation analysis. The specific form of the signal is as follows:

*s_x_*(*t*) = 0.05cos(2*πt* + *π*/3) (20)

As shown in Figure 4b, a noise signal *n*(*t*) with a suitable vibration amplitude is generated from a six-degree space vibration test bench. In the simulation, we still take the x-axis accelerometer signal as an example, and set its noise signal as follows:

*n_x_*(*t*) = (2 × 0.4)^1/2^*g*(*t*) (21)

where *g*(*t*) is a Gaussian white noise signal with a mean value of 0 and a variance of 1.

The frequency range of the noise signal *n_x_*(*t*) is between (0, 20)/(Hz), so it contains the part which is mixed with the frequency of signal *s_x_*(*t*). By superposing the measured signal and the noise signal, the noisy measurement signal *I*_x_(*t*) = *s_x_*(*t*) + *n_x_*(*t*) is obtained, which is substituted into the right end of Formula (1). At this time, the SNR of the x-axis accelerometer signal *I*_x_(*t*) is about −25 dB, which is consistent with the SNR condition of the actual attitude measurement.

The frequency detection model based on the SR of the Duffing system is constructed, and the fourth-order Runge Kutta algorithm is used to solve the model. The model parameters are set as follows: damping ratio *k* = 0.5, nonlinear coefficient *a* = *b* = 1, signal amplitude *λ* = 0.05, frequency *f*_0_ = 1 Hz, initial phase *φ* = π/3, sampling frequency *f*_s_ = 500 Hz, calculation step 0.002 s, and the data length *N* = 5000.

**Figure 5:**
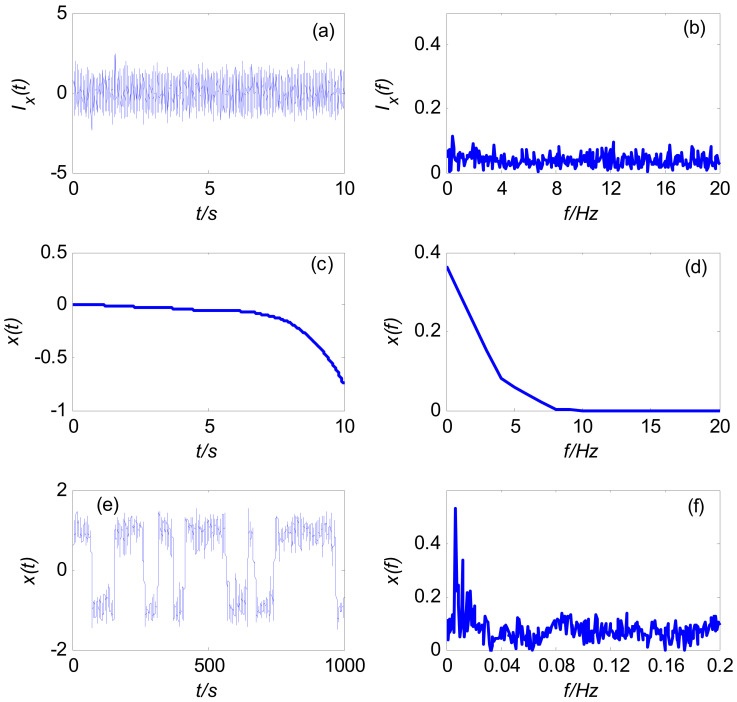
SR of the measurement signal with noise: (**a**) waveform of the input signal; (**b**) spectrum of the input signal; (**c**) waveform of the output signal; (**d**) spectrum of the output signal; (**e**) waveform of the output signal based on a variable scale; (**f**) spectrum of the output signal based on a variable scale.

Under the condition of large frequency parameters, the waveform and spectrum of the input signal of the Duffing system are shown in Figure 5a,b. From Figure 5a, it can be seen that due to the extremely low SNR of the input signal, the measured signal is annihilated in the noise signal, such that it is difficult to identify the useful signal *s_x_*(*t*) by direct spectrum analysis. From Figure 5b, it can be seen that the spectrum of the input signal of the Duffing system has no obvious peak at *f*_0_ = 1.

The waveform and spectrum of the system output signal *x*(*t*) are obtained by directly calculating Formula (1), as shown in Figure 5c,d. It can be seen from Figure 5d that the spectrum of the output signal of the Duffing system has no obvious peak feature at *f*_0_ = 1. This shows that the Duffing system frequency detection model has deviation under the condition of large frequency parameters, and there is no SR phenomenon in the direct numerical calculation, such that it is unable to identify the frequency of the useful signal in the noise. Therefore, it is necessary to use the frequency detection model based on the variable-scale Duffing system proposed in Section 2.2 to identify it.

For the large frequency parameters in Figure 5a, the variable-scale coefficient *R* = 100 is introduced in order to scale the measured signal, and it is then input into the Duffing system detection model to obtain the output signal waveform and spectrum, as shown in Figure 5e,f. At this time, the corresponding secondary sampling frequency *f*_sr_ = *f*_s_/*R* = 5 Hz, the calculation step is 0.2 s, and the simulation time is 1000 s. The maximum peak frequency obtained from the spectrum Figure 5f of the variable-scale output signal is 0.01 Hz, which is converted to the frequency of the original measured signal as 0.01 × *R* = 1 Hz, which is the frequency *f*_0_ of the measured signal *s_x_*(*t*). Therefore, the SR effect can be realized through the frequency/time scale transformation, in order to complete the frequency identification of the useful signal.

#### 4.1.2. Simulation Experiment of the Parameter Estimation Based on CPT

**Figure 6:**
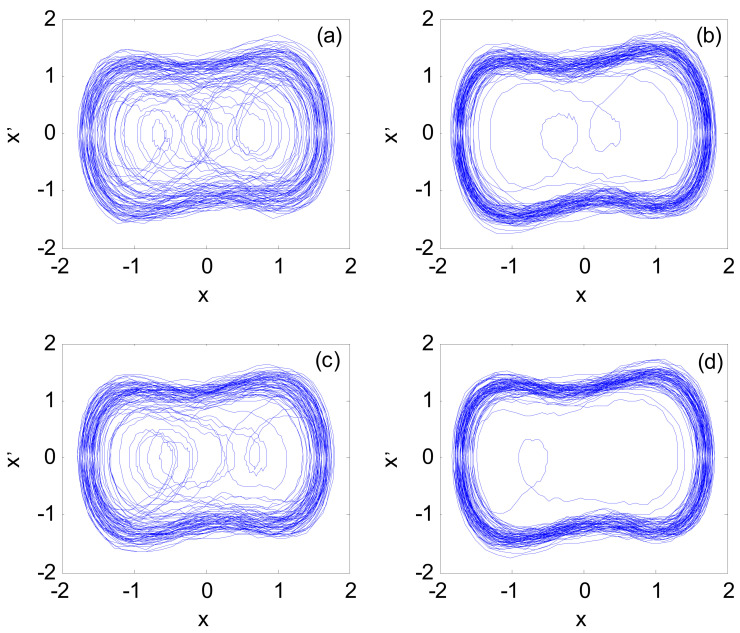
Phase space of the chaotic system: (**a**) while *α* = 0 and *A_a_*_1_ = 0.800; (**b**) while *α* = 0 and *A_a_*_1_ = 0.801; (**c**) while *α = π* and *A_a_*_2_ = 0.850; (**d**) while *α* = *π* and *A_a_*_2_ = 0.851.

After the frequency value of the signal *s_x_*(*t*) is determined, the Duffing chaos detection model is constructed with *I*_x_(*t*) as the input signal, and the amplitude and phase parameters of *s_x_*(*t*) are estimated. The parameters are set as follows: initial value x(0) = x′(0)= 0, driving signal amplitude *A* = 0.825, and the other parameters are the same as Section 4.1.1. The fourth-order Runge Kutta algorithm is still selected to solve the model, the calculation step is 0.02 s, the simulation time is 1000 s, and the data length is *N* = 50,000. The specific calculation steps are described in steps 5–7 of Section 3. The identification result of the *I*_x_(*t*) parameter is shown in Figure 6.

Figure 6a,b are the chaotic system phase space when *I*_x_(*t*) is input to the Duffing system with α = 0, and the driving signal amplitudes are 0.801 and 0.800, respectively. The observation shows that the amplitude of the system phase state jumping into a large-scale periodic state is 0.801, that is, *A_a_*_1_ = 0.801; Figure 6c,d are the system output phase diagrams when *I*_x_(*t*) is input to the Duffing system with α = π, and the driving signal amplitudes are 0.851 and 0.850, respectively. It is known that the amplitude of the phase transition to a large-scale periodic state is 0.851, that is, *A_a_*_2_ = 0.851. By substituting *A_a_*_1_ and *A_a_*_2_ into Equation (19), the amplitude and phase parameters of *s_x_*(*t*) become 0.048 and 58.4°, respectively.

The error statistics of the parameter estimation of the useful signal *s_x_*(*t*) are shown in Table 2.

According to the statistical results in Table 2, the accuracy of the amplitude and phase parameters obtained by the synchronous estimation method is high. The relative error of the input signal amplitude is about 4%, and the error of the initial phase is less than 3%.

#### 4.1.3. Analysis of the Attitude Angle Solving Results

In order to show the effectiveness of the method proposed in this paper, the attitude of the measured signal detected by SR and CPT based on a variable-scale Duffing system is calculated, and the results are compared with the original measured signal.

**Figure 7:**
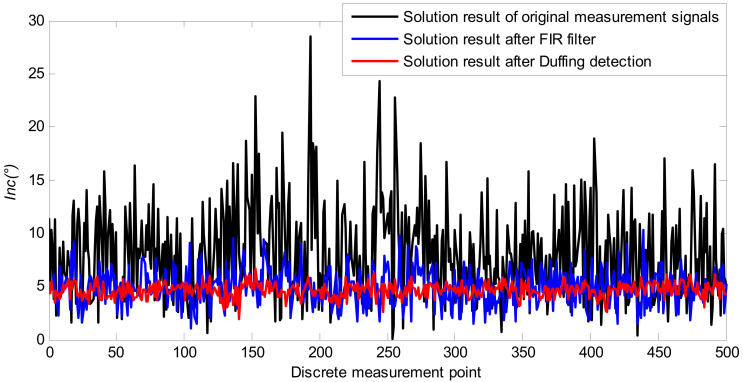
Comparison of the solution results for the inclination.

Firstly, the second-order Duffing system is used for the frequency detection and parameter estimation of a y-axis accelerometer signal in the same period. The complete detection process is the same as the x-axis input signal *I*_x_(*t*) in Section 4.1.1 and Section 4.1.2. The simulation results are similar to *I*_x_(*t*), which is not given here due to length limitation. Then, for comparison, FIR filtering is used to eliminate the noise of the two-axis accelerometer signals. Finally, the attitude of the measured signal processed by these two methods and the original measured signal is calculated, as shown in Equation (22), where *G* is the acceleration of gravity. During the simulation, the preset deviation angle is 4.5°, and the simulation results are shown in Figure 7.

(22) $$Inc=\arcsin\frac{\sqrt{I_{x}^{2}+I_{y}^{2}}}{G}$$

The simulation results show that the deviation angle obtained by directly calculating the attitude of the original measurement signal is quite different from the set value. In contrast, the measurement accuracy of the inclination can be effectively improved by filtering the original signal with FIR and then calculating the attitude; however, due to the high noise intensity, the SNR of the measured signal is only −25 dB, such that the filtering accuracy needs to be further improved.

On the other hand, the attitude solution of the measured signal after the cooperative detection of the SR and CPT of the second-order Duffing system has higher accuracy and is closer to the actual set value; in the case of extremely low SNR, the attitude solution result is relatively stable and there is no obvious error fluctuation. Therefore, the proposed method can obtain more accurate and more stable drilling tool attitude parameters under strong vibration and noise interference.

Further statistics show the detailed error results for the inclination, as shown in Table 3.

It can be seen from Table 3 that the root mean square (RMS) error and the maximum error of the inclination was only 0.68° and 2.53°, respectively, after the Duffing detection. This is not only far less than the attitude solution error of the original measured value but also better than the solution result of the FIR filter. Furthermore, the feasibility and validity of the proposed weak signal detection method were proven in laboratory testing.

### 4.2. Field-Drilling Testing

**Figure 8:**
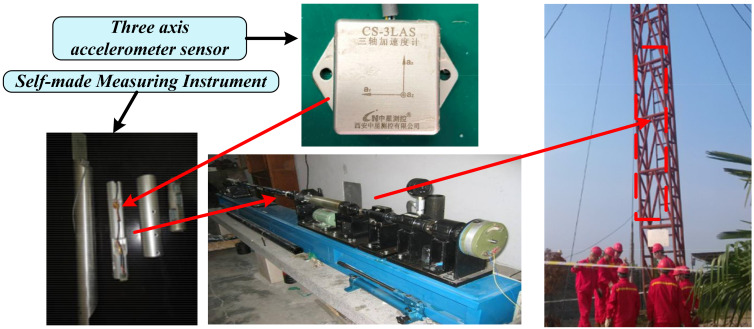
Schematic of the field test. The electronic circuit was installed in the axis of the drill collar near-bit, and the measurement data can be stored in real-time.

In order to further verify the feasibility and overall performance of the proposed detection method for a weak SNR signal, field-drilling data were used for testing and analysis. The experimental data came from the field-drilling process of a well in northern Shaanxi. The field acquisition process and installation position of the triaxial accelerometer sensors are shown in Figure 8 [2], and the drilling and production environment parameters are listed in Table 4. During drilling, the guiding tool was in a stable and straight state.

The CS-3LAS sensor, which was developed by the Zhongxing measurement and control company, was chosen as the accelerometer in this test. The accelerometer is suitable for the specific requirements of downhole drilling. The specific parameters of the sensors are shown in Table 5.

**Figure 9:**
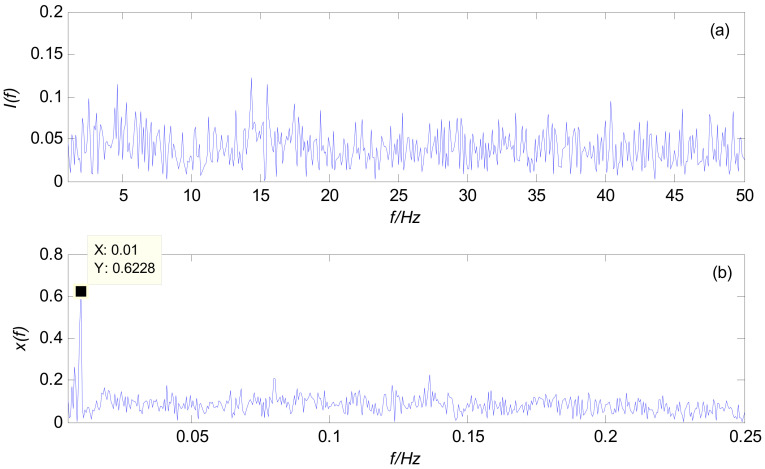
SR phenomenon of a field-drilling measured signal: (**a**) spectrum of the system input; (**b**) spectrum of the system output based on a variable scale.

In the same way as the simulation experiment, the measured signal of the x-axis accelerometer is taken as the experimental object and discretion. Firstly, the SR method of the variable-scale Duffing system is used to identify the frequency of a discrete data point, and the results are shown in Figure 9. It can be seen from Figure 9a that the spectrum range of the system input signal is very wide, and there is no obvious spectrum peak feature. This is mainly because, in the process of MWD, affected by various vibration effects, the spectrum of the noise presents the characteristics of a high frequency, a wide frequency, and randomness. At this time, it is almost impossible to detect the frequency by direct spectrum analysis.

According to the preset value of the rotary speed (*f* = 2 Hz) in the drilling process, the variable-scale coefficient *R* = 200 is introduced to transform the scale of the measured signal, and it is then input into the Duffing system detection model. Due to the influence of strong vibration, high temperature, high pressure, and other factors, there is a certain error between the actual rotation speed and the preset value. Therefore, in the detection, the variable-scale coefficient needs to be adjusted up and down based on *R* = 200. Finally, the spectrum of the output signal when *R* = 189 is obtained, as shown in Figure 9b. It can be seen that the maximum spectral peak frequency obtained from the spectrum of the variable-scale output signal is 0.01 Hz, and the frequency converted to the original signal to be measured is 0.01 × 189 = 1.89 Hz, which indicates that the characteristic frequency *f*_0_ of the output signal is 1.89 Hz at this time, in order to complete the frequency identification of the measured signal.

**Figure 10:**
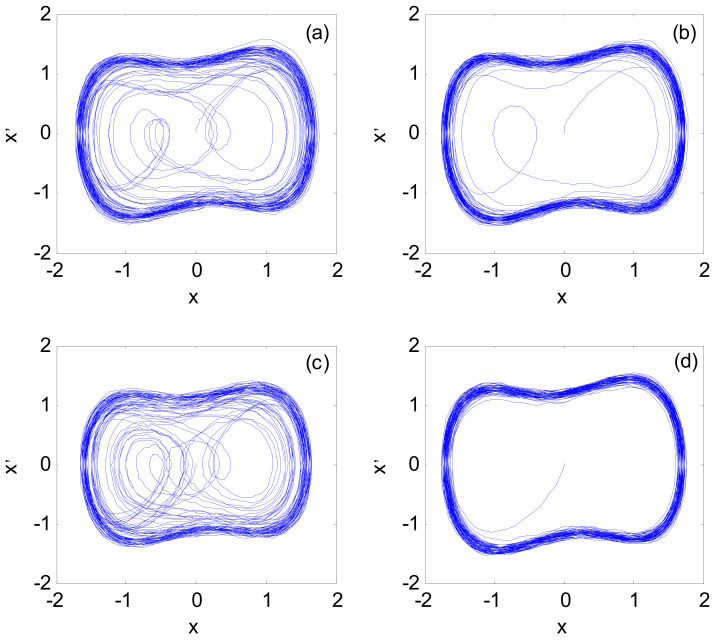
Phase space of a chaotic system: (**a**) while *α* = 0 and *A_a_*_1_ = 0.8367; (**b**) while *α* = 0 and *A_a_*_1_ = 0.8368; (**c**) while *α = π* and *A_a_*_2_ = 0.8167; (**d**) while *α* = *π* and *A_a_*_2_ = 0.8168.

After the frequency value of the x-axis accelerometer signal is determined, the amplitude and phase parameters are estimated by CPT based on the Duffing chaotic model. The parameter setting and calculation process are the same as the simulation experiment in Section 4.1.2. The parameter identification results of the discrete X-axis accelerometer measurement signal are shown in Figure 10.

When the initial phase α of the driving signal is 0 and the amplitudes are 0.8367 and 0.8368, respectively, the phase space is shown in Figure 10a,b; it can be seen that the amplitude of the system phase state jumping into the large-scale periodic state is at 0.8368, that is, *A_a_*_1_ = 0.8368; when the initial phase α of the driving signal is π and the amplitudes are 0.8167 and 0.8168, respectively, the phase space is shown in Figure 10c,d; it can be seen that the system phase state is jumping. The amplitude of the large-scale periodic state is at 0.8168, that is, *A_a_*_2_ = 0.8168. By substituting *A_a_*_1_ and *A_a_*_2_ into Equation (19), the amplitude and phase parameters of the measured signal become 0.021 and 118.8°, respectively.

It should be pointed out that the smaller the amplitude of the measured signal, the more accurate the amplitude of the driving signal. For the field-drilling signal, if the amplitude of the driving signal is still changed by the amplitude of 0.001 in the simulation test, the accurate parameter estimation cannot be obtained. For this reason, the amplitude of the driving signal is increased or decreased by 0.0001 in the field-drilling test, and the more accurate parameter estimation is finally obtained; however, this will increase the calculation time of the algorithm and affect the real-time performance of MWD. Therefore, in practical application, when the amplitude of the measured signal is very low, it is necessary to strike a balance between the parameter estimation accuracy and the calculation time, and to select the appropriate amplitude interval according to the specific requirements of the down-hole actual measurement.

**Figure 11:**
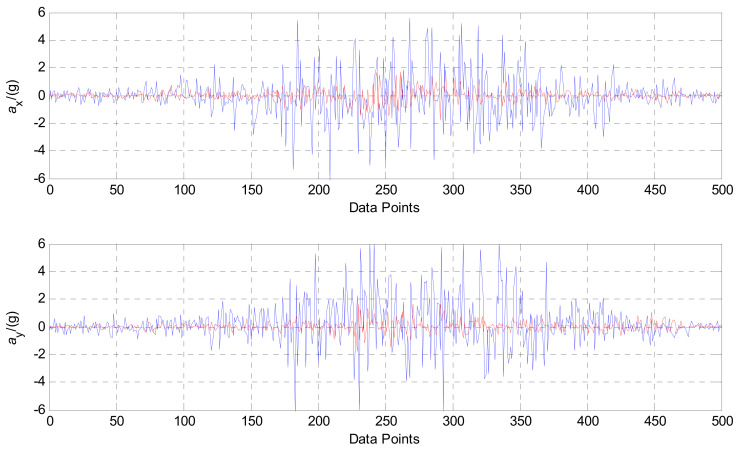
The estimation results of the field-drilling signal of the x and y-axis accelerometer sensor by SR and CPT based on the Duffing system.

In the same way, frequency identification and parameter estimation are performed for 500 discrete x-axis and y-axis measurement points. The measurement results after the signal recovery are drawn and compared with the original measurement signals. The comparison results are shown in Figure 11. We cannot estimate the error of this process, because the data came from the field test, for which exact measurements are impossible to determine. However, it is evident that the fluctuation amplitude of each piece of accelerometer sensor data, which was processed by SR and CPT based on the Duffing detective system, was significantly smaller. This showed better noise anti-interference ability.

**Figure 12:**
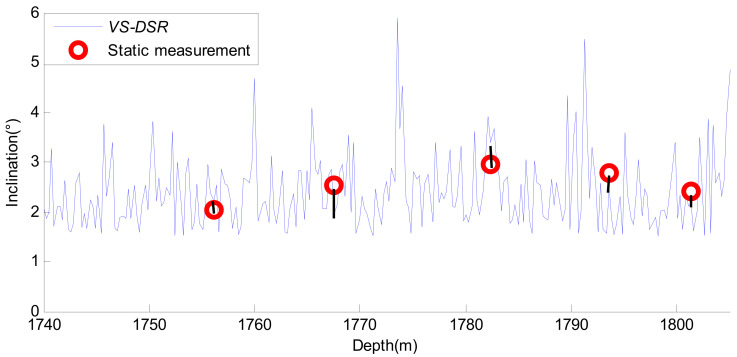
Solving the result of the inclination at northern Shaanxi.

In order to further evaluate the overall performance of the proposed method, the data after the Duffing detective system were used to obtain the real-time attitude parameters of the drilling tool. There was no interference of vibration acceleration in the attitude measurement of the stopping of the drilling, which ensures the accuracy of the attitude parameters, such as inclination. Therefore, it was used as a reference value to verify the performance of the SR and CPT based on the Duffing detective method. The solution results of the inclination obtained by the proposed method are given in Figure 12. As a comparison, the static measurement results of five data points are given in Figure 12. The black bar in Figure 12 shows the difference between the static measurement and the actual measurement results. It can be seen that the static measurement results of the five data points are close to those obtained by the method proposed in this paper.

Based on Figure 12, further statistics show the detailed error results for the inclination, as shown in Table 6. As a comparison, the measurement results of five data points after FIR filtering are given in Table 6.

It can be seen from Table 6 that the overall solution accuracy of the inclination field drilling data is lower than that of the simulation data. This is mainly because of the process of drilling, wherein the measured signal is interfered with by many factors, including server vibration, drill string rotation, high temperature, and pressure, etc. On the other hand, vibration interference is not an ideal Gaussian white noise signal in the process of drilling. All of these will affect the result of the filtering.

It can also be concluded from Table 6 that the relative error of the drilling tool inclination after it is processed by the proposed method is almost 10%. The maximum relative error is 23.32% from the Duffing cooperative method, but it has only one data point. Compared with FIR, the accuracy of the attitude solution is improved, and the error fluctuation amplitude is significantly reduced. This shows that the proposed method in this paper provides a new solution to the detection problem of weak SNR signals during MWD.

## 5. Conclusions

In the process of oil and gas well drilling, there are multi-frequency and high amplitude interference signals in attitude measurement due to severe vibration and rapid rotation. In order to solve this problem, this paper proposes a method of the frequency detection and parameter estimation of weak SNR signals while drilling based on the SR and CPT cooperative of a variable-scale Duffing system. The conclusions are summarized as follows: 

(1) The proposed SR detection algorithm based on a variable-scale Duffing system can complete the parameter reconstruction of the frequency value of the measured signal without changing the discrete value, and can meet the small frequency parameter requirements of SR detection of the Duffing system. The experimental results show that the method is feasible and effective.

(2) Two Duffing oscillators with a different initial phase of driving signal are combined in order to estimate the amplitude and phase parameters of the measured signal. The experimental results show that the method can detect the amplitude and phase of the signal synchronously in severe background noise, and the estimation accuracy is high, which can effectively improve the accuracy of the attitude angle of the drilling tool.

(3) The proposed detection algorithm is used to identify the stimulation signal with an amplitude of 0.05, and the results show that the SNR of the method is as low as −19 dB; the experimental results show that with the decrease of the amplitude of the signal to be detected, the SNR of the signal to be detected is further reduced. The algorithm proposed in this paper provides a solution to the problems of weak SNR signals in MWD.

In addition, the theoretical analysis and simulation analysis show that the proposed method also has some limitations in its application, which are mainly reflected in the following two aspects: (1) due to the influence of parameter setting (the influence of noise intensity in SR) and operation state judgment, there are still some shortcomings, such as the low detection efficiency and easy misjudgment of the state transition; (2) each detection needs to complete the construction of two models (SR and CPT), and it is difficult to deal with the tested object with multiple frequency signals, which affects the applicability of the proposed method. 

Therefore, future research studies will be carried out with regard to the following aspects: (1) we should not be limited to the Duffing oscillator as a nonlinear system, and should consider other systems that can produce stochastic resonance or chaos, such as Lorenz system or Chua’s circuit, and identify the frequency of the measured signal through its chaotic synchronization phenomenon; (2) we should combine the nonlinear system with other methods (such as neural networks) to further improve the detection efficiency and applicability.

## Figures and Tables

**Table 1:** The characteristics of the vibration signal in MWD.

Vibration Type	Main Modes	Frequency Range (Hz)
Stick-Slip	Torsional Vibration	0.01–5
Bounce	Axial Vibration	1–10
Rotate	Lateral Vibration	3–50
Lateral Shock	Lateral Vibration	Irregular
Torsional Resonance	Torsional Vibration	20–350
Parametric Resonance	Axial and Lateral	0.1–10
Modes Coupled	Axial, Lateral, and Torsional	0.1–20

**Table 2:** Estimation results of the amplitude and phase of the useful signal.

Input Signal	*A_a_*_1_	*A_a_*_2_	Amplitude Estimation Result	The Relative Error of Amplitude	Phase Estimation Result	The Relative Error of Phase
sx(t)	0.801	0.851	0.048	4%	58.4°	2.7%

**Table 3:** Error statistics of the attitude solution.

Data Processing Method	Attitude Parameter	Statistical Index of Solution Error	
		Max Error	RMS Error
SR and CPT based on Duffing system	Inclination (°)	2.53	0.68
FIR filter	Inclination (°)	5.78	1.73
Original measurement data	Inclination (°)	24.01	5.54

**Table 4:** Basic parameters of the field-drilling test.

Parameters	Value
Well depth	1740–1805 m
WOB	10 MPa
Downhole temperature	40 °C
Pump pressure	6.6 MPa
Drilling fluid density	1.15 g/cm^3^
Suspended load	79 kN
Operation time	75 h
Rotary speed	120 rpm
The setting value of inclination	2.5°

**Table 5:** Characteristics of the accelerometer sensors.

**The Performance Index**	Axial	X	Y	Z
	Range	±3 g (±1~±100 g)		
	Bandwidth	0 to ≥500 Hz		
	Scale factor	300 ± 30 mV/g		
	Calibration	≤1 mg		
	Non-linearity	≤0.3% Fs		
	Zero-bias (25 °C)	1.5 ± 0.1 V		
	Zero-bias temperature drift	±1 mg/°C		
	Startup time	≤0.001 s		
**Environmental Characteristics**	Work temperature	−40 °C ~ +70 °C		
	Storage temperature	−40 °C ~ +125 °C		
	Anti-crash (0.5 ms)	10^4^ g		
**Physical Characteristics**	Weight	40 g		
	Size	19.5 × 18 × 10 mm		

**Table 6:** Error statistics of the attitude solution.

Algorithm	Attitude Parameter	Depth/m				
		1756.12	1767.56	1782.38	1793.56	1801.36
Static Measurement	Inclination (°)	2.04	2.53	2.95	2.77	2.41
FIR filter	Inclination (°)	2.50	2.17	2.41	1.93	3.12
	Relative error	22.55%	14.23%	16.61%	30.32%	29.46%
SR and CPT based on Duffing system	Inclination (°)	2.20	1.94	3.39	2.51	2.17
	Relative error	7.84%	23.32%	12.54%	9.39%	9.96%

## Data Availability

Some or all data, models, or code generated or used during the study are proprietary or confidential in nature and may only be provided with restrictions.
